# Spectrum of hepatofugal collateral pathways in portal hypertension: an illustrated radiological review

**DOI:** 10.1007/s13244-015-0419-8

**Published:** 2015-09-04

**Authors:** Ankur Arora, S. Rajesh, Yamini S. Meenakshi, Binit Sureka, Kalpana Bansal, Shiv Kumar Sarin

**Affiliations:** Department of Radiology, Institute of Liver & Biliary Sciences, D-1 Vasant Kunj, New Delhi, 110070 India; Department of Hepatology, Institute of Liver & Biliary Sciences, D-1, Vasant Kunj, New Delhi, 110070 India

**Keywords:** Collateral pathways, Multi-detector row computed tomography, Portal hypertension, Shunt, Varices

## Abstract

The purpose of this article is to describe the various portosystemic collateral pathways pertinent to portal hypertension on multi-detector row computed tomography (MDCT) and their clinical relevance, with special emphasis on the uncommon ones. The knowledge and understanding of the various patterns of portosystemic collateral channels has important implications both for the clinician and the interventionist. MDCT with its advanced post processing capabilities can exquisitely demonstrate these vascular pathways to help in therapeutic decision making.

*Teaching points*

*• Portosystemic collaterals are an important cause of bleeding and hepatic encephalopathy.*

*• Radiologists should be familiar with the imaging findings to effectively identify them.*

*• Pre-operative knowledge of portosystemic collaterals is essential to avoid inadvertent vascular injury.*

## Introduction

Portal hypertension (PHTN), characterized by a pathological increase in the portal venous pressure, is one of the key consequences of liver cirrhosis [1]. It results from a combination of increased intrahepatic vascular resistance and augmented blood flow through the portal venous system [1]. This high-pressure hepatopetal flow is redirected through alternative pathways into the low-pressure systemic veins, leading to formation of an extensive network of portosystemic collateral vessels (PSCV) [2]. Detection of these ‘spontaneous’ PSCV serves as an important tool in diagnosing portal hypertension and predicting prognosis [3]. The radiological appearances of the *common* PSCV, including gastro-oesophageal and para-oesophageal collaterals, gastrorenal or splenorenal shunts, and paraumbilical shunts have been studied at length [4–8]. However, with the advent of multi-detector row computed tomography (CT), unusual pathways of portosystemic anastomoses are increasingly being recognized, yet have not been well described in the literature [9–14]. Since these shunts could be an important cause of variceal bleeding and hepatic encephalopathy, their accurate identification is imperative in therapeutic decision making. In addition, understanding their anatomy may help to avoid potential complications related to interventional radiological procedures and surgery. The purpose of this review is to appraise the spectrum of common and uncommon collateral pathways of the portal venous system that can be encountered in PHTN at CT examinations.

Embryologically derived anastomoses between the portal and systemic circulation exist at various sites in normal healthy humans [3] (Table 1).

**Table 1:** Normal sites of portosystemic anastomoses

Sites	Portal component	Systemic component
Lower oesophagus	Left gastric vein	Oesophageal veins
Rectum and anal canal	Superior rectal vein	Middle and inferior rectal veins
Umbilicus	Paraumbilical veins	Superior and inferior epigastric veins
Bare area of liver	Portal venous branches	Inferior phrenic and right internal thoracic vein
Retroperitoneum	Tributaries of splenic and pancreatic and colic veins	Renal, suprarenal, paravertebral and gonadal vein
Patent ductus venosus (rare)	Left branch of portal vein	Inferior vena cava (IVC)

**Fig. 1:**
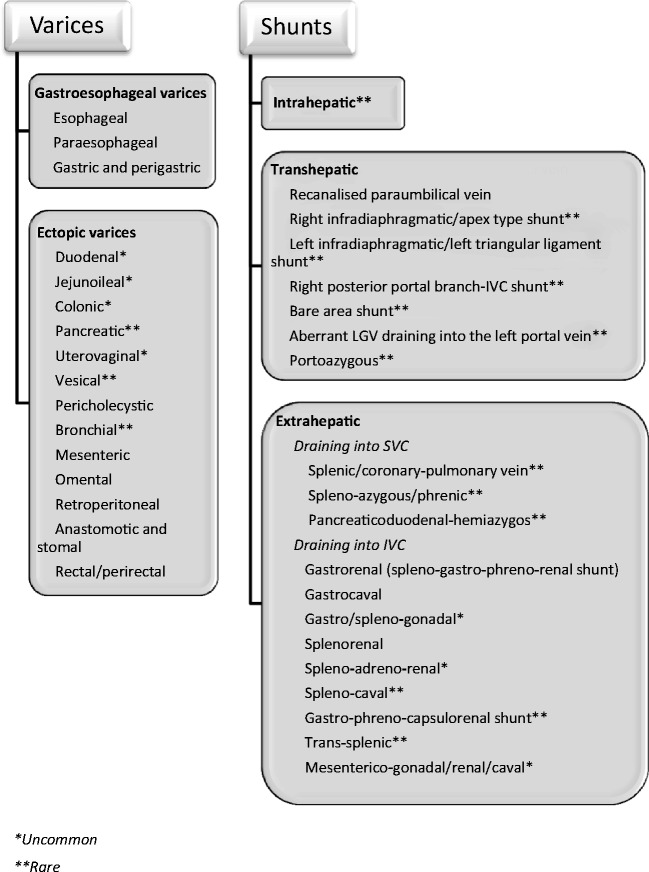
Portosystemic collateral pathways described in English-language medical literature

In PHTN, dilatation of these channels leads to formation of varices at various sites in the body, which are mostly classified into two groups, the gastro-oesophageal varices and ectopic varices [3] (Fig. 1). These varices are fed in the long term by spontaneous development of large shunts in the abdomen, which can be anatomically divided into intrahepatic, transhepatic and extrahepatic shunts [9–12] (Fig. 1).

## Varices

### Oesophageal and para-oesophageal varices

**Fig. 2:**
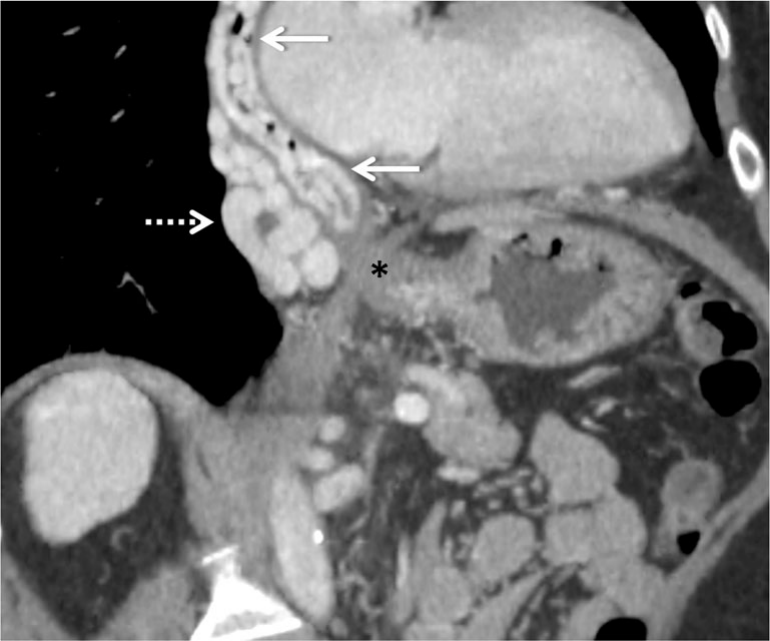
Coronal-oblique maximum intensity projection (MIP) contrast-enhanced CT (CECT) image demonstrating multiple collaterals in the oesophageal mucosa (*solid arrows*) and para-oesophageal region (*interrupted arrow*). *Asterisk* denotes the gastro-oesophageal junction

**Fig. 3:**
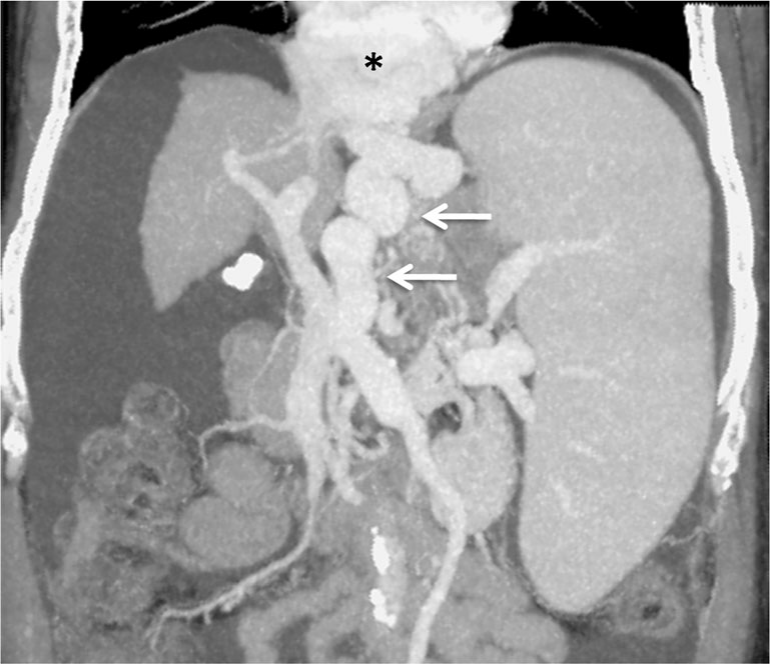
.Coronal MIP image showing a bunch of para-oesophageal collaterals (*asterisk*) being supplied by a dilated left gastric vein (*arrows*)

Oesophageal varices are detected in about 50 % of patients with liver cirrhosis and approximately 5–15 % of cirrhotics show newly formed varices or worsening of varices each year [15–17]. The term *oesophageal varices* usually refers to the dilated veins located within the wall of the lower oesophagus, whereas *para-oesophageal varices* are situated outside the wall of the oesophagus (Fig. 2). Oesophageal varices are usually supplied by the anterior branch of the left gastric vein, whereas the posterior branch of this vein directly forms the para-oesophageal collateral vessels [6] (Fig. 3).

**Fig. 4:**
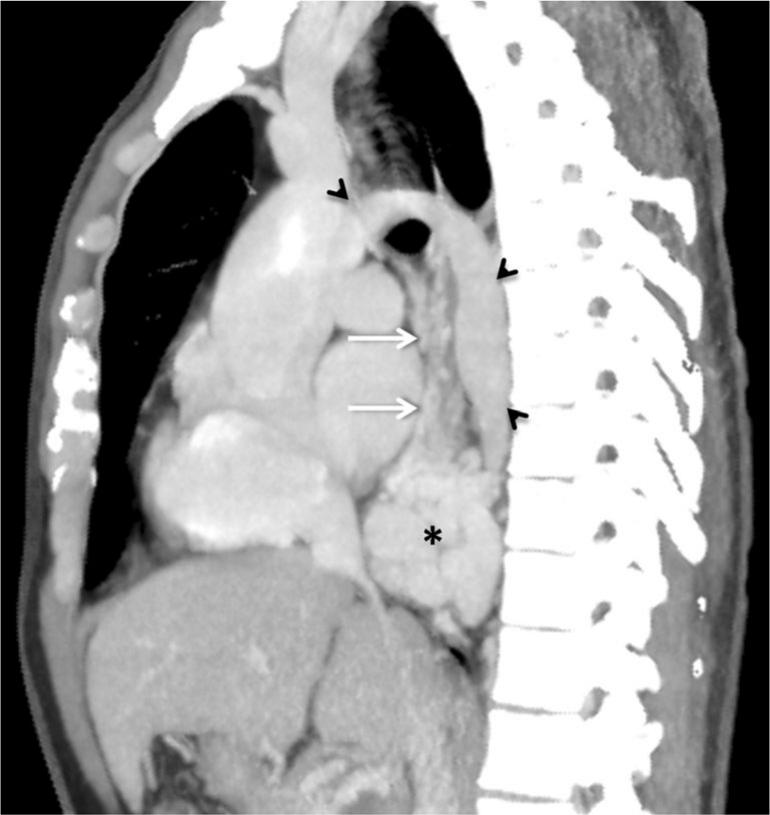
Sagittal MIP image from another patient demonstrating multiple oesophageal and para-oesophageal collaterals (*arrows* and *asterisk*, respectively) drained via dilated azygous vein (*arrowheads*)

Blood from the oesophageal varices usually drains into the left subclavian vein and/or brachiocephalic vein, while the blood of the para-oesophageal varices commonly drains into the azygos or hemiazygos system (Fig. 4). The oesophageal or para-oesophageal varix may rarely drain into the inferior vena cava (IVC) [3, 5].

Oesophageal varices are the most common to bleed in cirrhotic patients, owing to the high-volume of blood carried by them, and account for the high mortality associated with spontaneous variceal bleeding [16].

### Gastric and perigastric varices

**Fig. 5:**
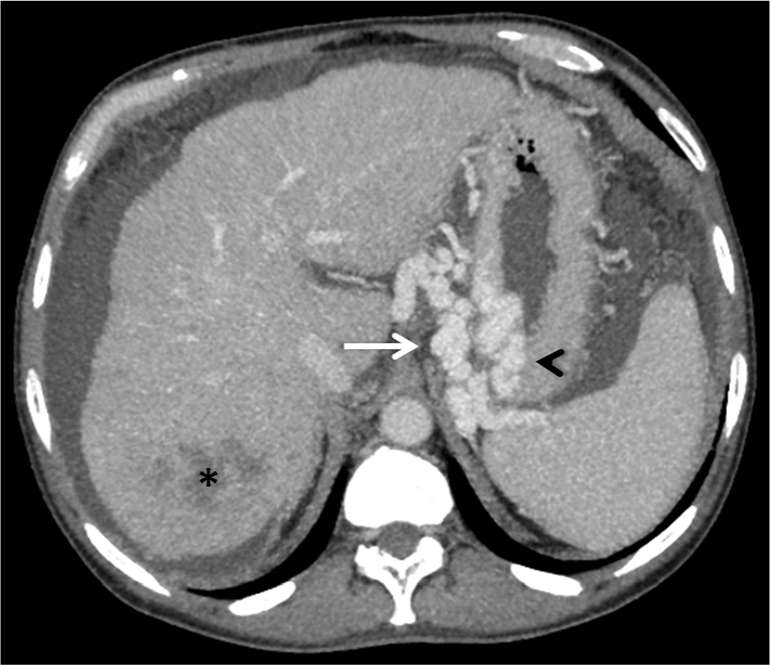
Axial CECT image showing multiple collaterals in the gastric mucosa (*arrowhead*) and perigastric region (*arrow*). The patient also had a right lobe hepatocellular carcinoma (*asterisk*)

**Fig. 6:**
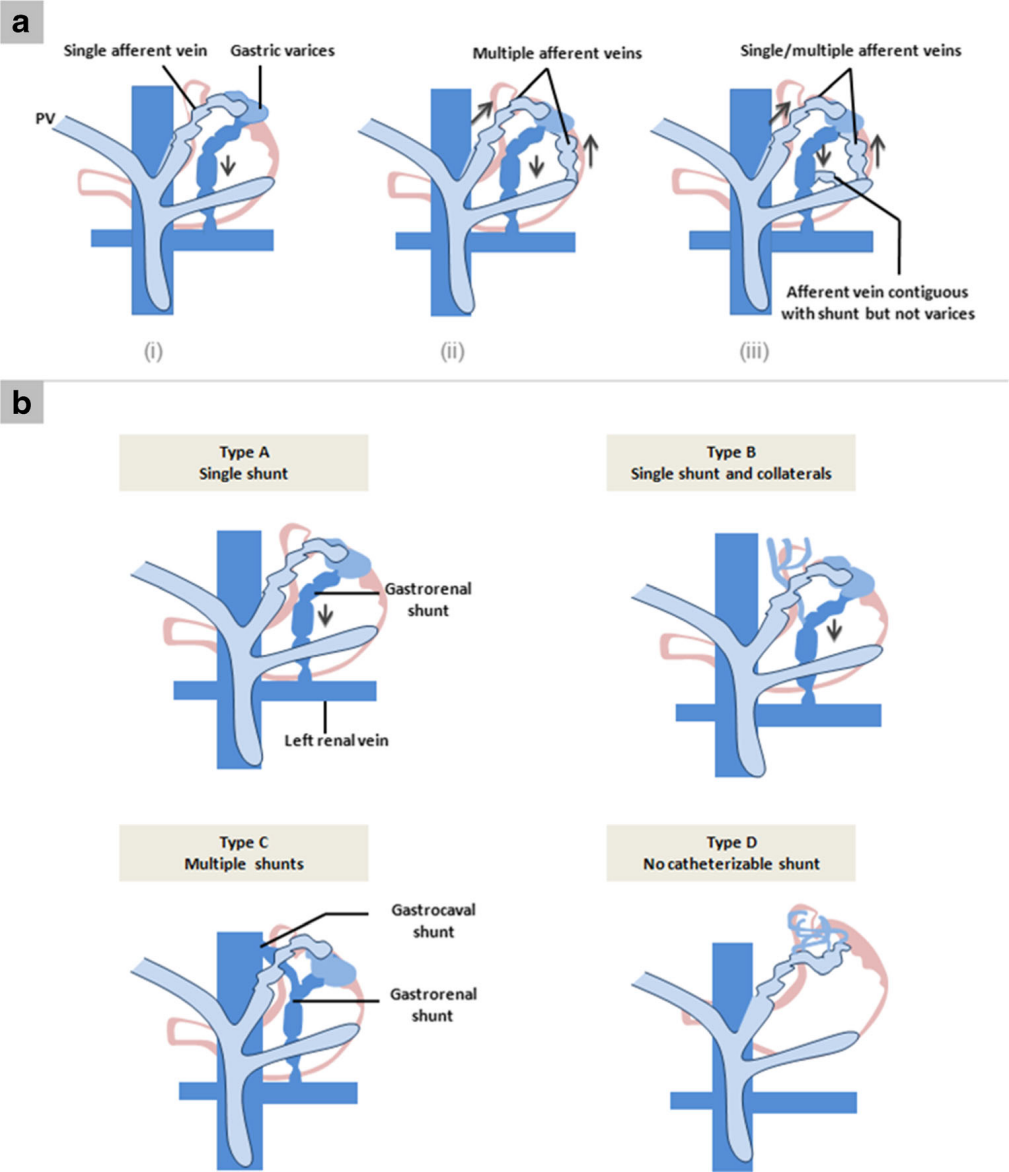
Graphic illustration of the Kiyosue classification of GV [21]. **a** *Classification based on the inflow pathway(s)*. Type 1 (i) refers to a single afferent vein for the varices, while type 2 (ii) refers to multiple afferent vessels contributing to the varix. Type 3 is like type 2 with the addition of small afferent vein(s) in direct continuity with the outflow tract. **b** *Classification based on drainage pathway*. Type A consists of a gastrorenal shunt as the sole drainage. Type B describes the additional presence of small portosystemic collaterals; type C describes the presence of both a gastrorenal and direct gastrocaval shunt; type D consists of multiple small portosystemic collaterals as the drainage pathways without any recognisable shunt

Gastric varices (GV) (Fig. 5) are less prevalent than oesophageal varices, being present in 5 to 33 % of patients with portal hypertension. They have a reported incidence of bleeding of about 25 % in 2 years [18–20]. They are classified and graded on endoscopy by various authors. Currently, the most commonly used classification is the Sarin’s classification [18], which divides the GV into gastro-oesophageal (GOV) and isolated gastric varices (IGV). GOV are basically oesophageal varices that extend beyond the oesophagogastric junction and are further subdivided into GOV1, which extend for 2–5 cm along the lesser curvature, and GOV2, which extend along the fundus. IGV, as the name suggests, are not associated with oesophageal varices and are divided into IGV1, which are located in the fundus, and IGV2, present anywhere other than the fundus including the body, antrum or pylorus. GV are supplied by a single or multiple afferent gastric veins, commonly the left and posterior gastric veins, but are also seen with the short gastric veins, and rarely the gastroepiploic vein, which typically supplies the varices after endovascular or surgical exclusion of other main afferent veins [3] (Fig. 6). There are usually several short gastric veins that course along the greater curvature on the medial side of the spleen to empty into the splenic vein. [3] The posterior gastric vein is a distinct vein localised between the left and short gastric veins that runs superiorly in the retroperitoneum and gastrophrenic ligament and joins GV . GOV1 are formed by the anterior branch of the left gastric vein, and penetrate the gastric wall at the level of the cardia. GOV2 and IGV1 are usually fed by the short gastric and posterior gastric veins and commonly drain into the oesophageal or para-oesophageal veins (approximately 84 %) [3]. They may also drain into the  left renal vein by way of gastrorenal shunt, or directly into the IVC through a gastrocaval shunt via the left inferior phrenic and pericardiophrenic vein [3, 21, 22] (Fig. 6). Other smaller venous pathways include ascending lumbar vein, perivertebral venous plexus, intercostal veins, and rarely, the azygos vein [3].

The risk of bleeding from gastric varices is known to be lower than that from oesophageal varices, however, the severity of bleeding and the associated mortality is significantly higher, because of their large size and rapid blood flow [23]. Various treatment modalities, such as pharmacotherapy, balloon tamponade, endoscopic procedures, endovascular treatment, and surgery, have been used in their management [24–26]. For patients in whom endoscopic therapy fails to control the GV bleed or who re-bleed, interventional radiological techniques such as transjugular intrahepatic portosystemic shunt (TIPS), balloon-occluded retrograde obliteration of varices (B-RTO) or percutaneous transvenous embolisation (PTVE) of varices can be done [20, 23, 25]. In majority of the cases, the anatomy of varices dictates the approach used for treatment. Familiarity with the afferent and efferent veins is of paramount importance, as the degree of difficulty in performing endovascular obliteration of gastric varices and the success of the procedure are directly correlated with the anatomic complexity of the varix .

### Ectopic varices

Ectopic varices account for 2–5 % of gastrointestinal tract variceal bleeding [27–29]. However, they have a fourfold increased risk of bleeding when compared with oesophageal varices, and can have a mortality rate as high as 40 % [28–32].

Ectopic varices can either be a result of global portal hypertension or splanchnic venous occlusion. These occlusions can be due to thrombosis of the main portal vein, splenic vein, mesenteric veins or of a spontaneous gastrorenal shunt (post B-RTO) [7]. The occlusion can also be due to postoperative adhesions, scarring, and postoperative-altered anatomy [27, 31].

The standard management of ectopic varices has not yet been established. However, it is known that when bleeding occurs from ectopic varices, it is difficult to control by any means, and the bleeding is potentially fatal. All treatment strategies and techniques have been utilised in their management, including medical (systemic vasopressin and octreotide) and endoscopic therapy (banding/ligation and injection therapy), decompression using TIPS and partial splenic artery embolisation, antegrade/retrograde obliteration and surgical ligation [27–30, 32–35]. However, all of them have shown poor outcomes, underlining the importance of early diagnosis and therapy of these varices. Better understanding of ectopic varices is needed for a more systemic approach to this rare but menacing problem.

**Fig. 7:**
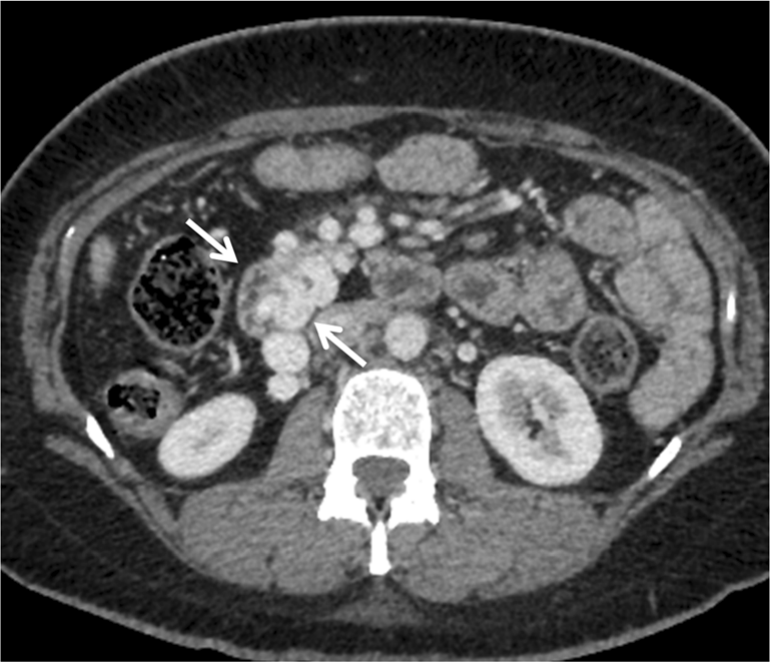
Axial CECT image showing multiple submucosal as well as paraduodenal collaterals along the third part of duodenum (*arrows*)

Duodenal varices (DV) resulting from intrahepatic portal hypertension are rather uncommon accounting for only 1–3 % of all cases [3]. The most common locations of duodenal varices are in the first and second portions of the duodenum (Fig. 7), although they can also be rarely seen in the distal duodenum [7]. The rare occurrence of bleeding from DV, in contrast to oesophageal varices, may be related to their smaller diameter, shorter length and deeper location on the outer wall of the duodenum [3].

The afferent vessel can be formed by any of the tributaries of portal venous system and commonly include the superior and inferior pancreaticoduodenal veins, cystic branches of the superior mesenteric veins, gastroduodenal vein, and pyloric vein [3, 7]. The efferents flow hepatofugally via retroperitoneal shunts (also called veins of Retzius) into the IVC via the right gonadal veins (mesenterico-gonadal shunt) or the capsular renal veins (mesenterico-renal shunt) [3, 7].

**Fig. 8:**
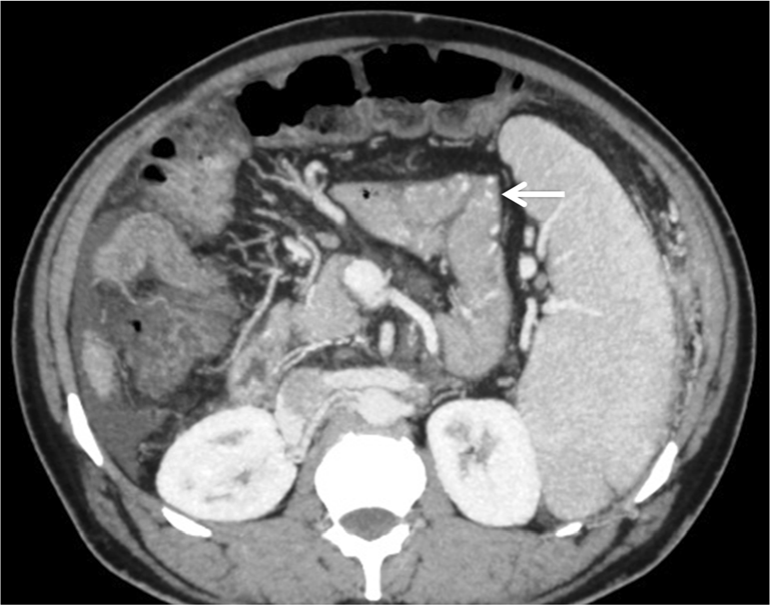
Axial CECT image showing multiple jejunal collaterals (*arrow*)

Jejunal and ileal varices are frequently associated with prior abdominal surgery [3]. The development of these varices is often due to collateral circulation through postoperative adhesions between the jejunum or ileum and the abdominal wall. Adhesions tend to bring the parietal surface of the viscera in contact with the abdominal wall, and portal hypertension results in the formation of varices [3, 7]. However, they can also be found in portal hypertensive patients without any prior history of surgical interventions (Fig. 8). The afferent vessels include the jejunal and ileal veins (tributaries of superior mesenteric vein) and the efferents generally drain into abdominal wall or the veins of Retzius [3].

**Fig. 9:**
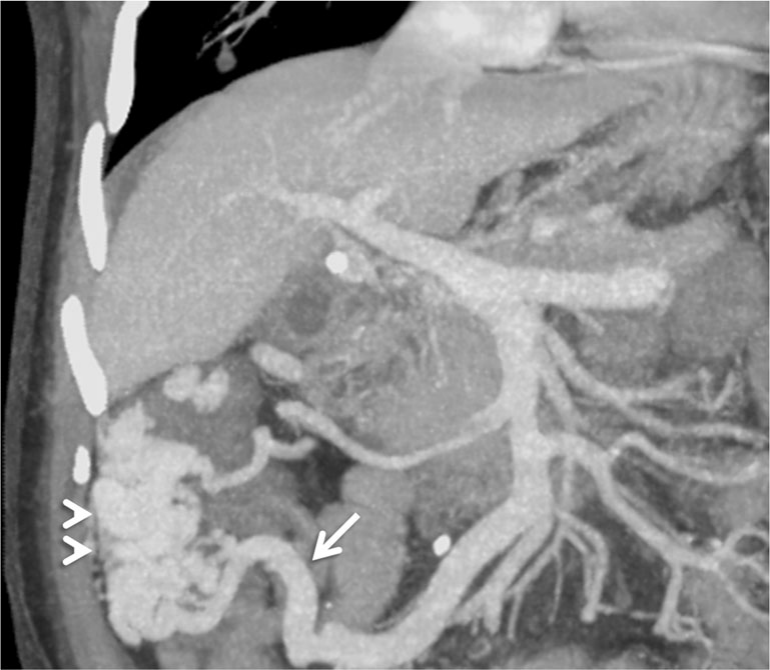
Coronal MIP image demonstrating multiple pericolonic collaterals (*arrowheads*) deriving their afferent supply from the superior mesenteric vein (*arrow*)

Colonic varices are usually found in a segmental distribution, primarily located in the cecum and the recto-sigmoid region [3] (Fig. 9). Although rectal varices are a common finding at colonoscopy, isolated varices of the colon are rare [36]. It has been hypothesised that colonic varices due to portal hypertension arise in patients in whom normal embryological colonic anastomoses are highly developed [36]. The afferent vessels include the ileo-colic, right, middle colic or sigmoid colic vein. Efferent veins include the right gonadal vein, right renal vein and systemic lumbar veins [3]. Recognition of this condition is important, as colonic varices may be an infrequent cause of massive lower gastrointestinal bleeding [36, 37]. Although there are reports of successful endoscopic therapy and TIPS, the treatment of colonic varices is not well defined [36, 37].

#### *Pancreatic varices*

**Fig. 10:**
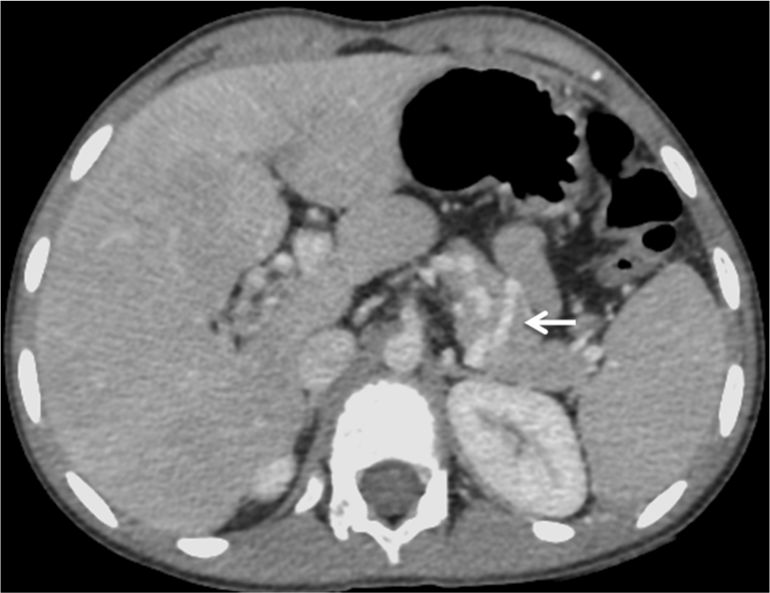
Axial CECT image showing collaterals in the pancreatic parenchyma (*arrow*) in a patient with portal vein thrombosis

Pancreatic varices are rare. When present, they are almost always associated with portal vein thrombosis with concomitant thrombosis of the splenic and the superior mesenteric veins (Fig. 10).

#### *Uterovaginal varices*

**Fig. 11:**
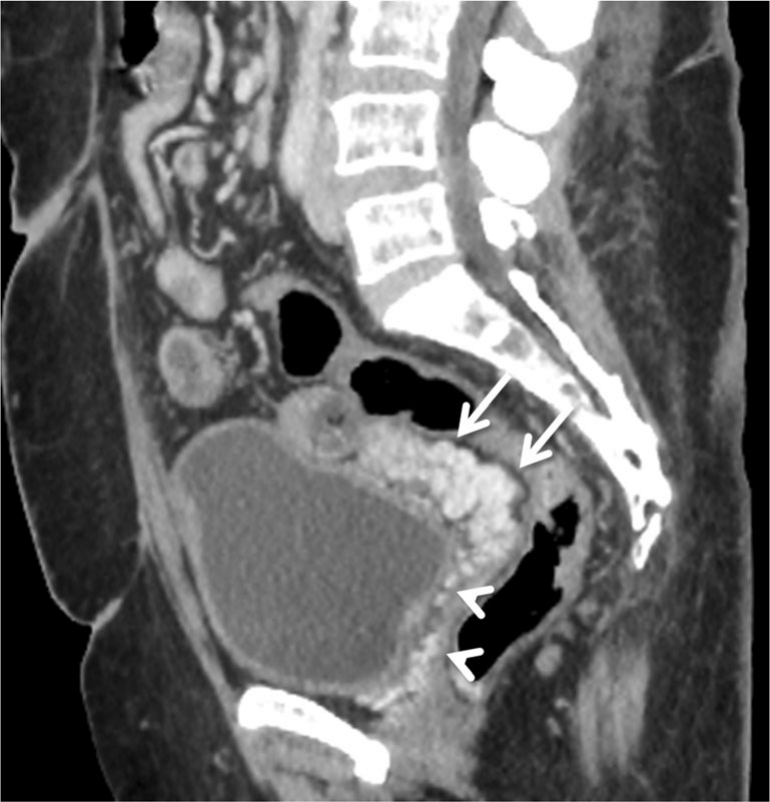
Sagittal MIP image showing multiple uterine and vaginal collaterals (*arrows* and *arrowheads*, respectively)

The uterus and vagina have an extensive network of venous channels that primarily drain into the systemic circulation via the uterine and hypogastric veins. The only communication between this plexus and the portal system is through the superior portion of the haemorrhoidal plexus. While anorectal varices are quite commonly found in cirrhotics, the extensive uterovaginal venous plexus ensures that the effects of portal hypertension are effectively decompressed without the formation of varices [38]. Thus, uterovaginal varices (Fig. 11) are exceptionally rare. To date, there have been only eight reported cases of vaginal variceal haemorrhage [39]. Barring one instance, all these cases occurred in patients who had previously undergone hysterectomy, leading to speculation that loss of the uterine venous plexus due to the surgery might be leading to venous congestion in vagina [40]. Massive haemorrhage has been reported to occur from vaginal varices that had to be controlled using suture ligation, banding, or sclerotherapy, together with local tamponade. TIPS has also been reported to be beneficial as a temporizing measure in reducing variceal pressure [38]. However, liver transplantation remains the definitive treatment [38].

#### *Vesical varices*

**Fig. 12:**
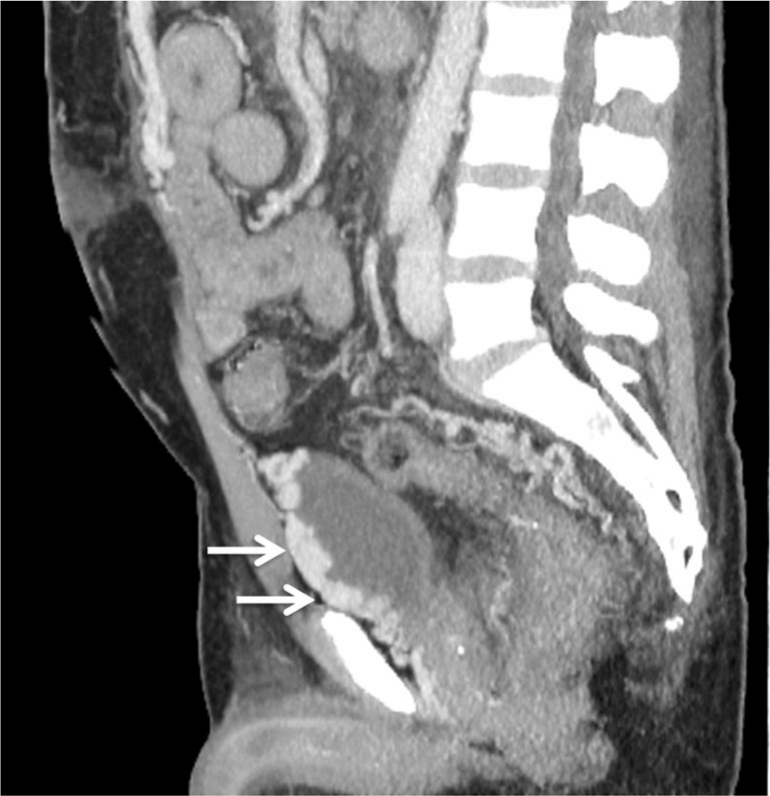
Sagittal MIP image showing multiple collaterals in the wall of the urinary bladder (*arrows*)

Vesical varices secondary to portal hypertension are extremely rare, with only a handful of reported cases, since the bladder wall is an unusual collateral route for the venous splanchnic blood [41–43]. They may appear when the usual splanchnic-bed collaterals cannot develop, thus allowing venous blood to flow through the venous system of the bladder [41] (Fig. 12). Generally reported cases of vesical varices have a history of abdominal surgery or intervention in the form of sclerotherapy and band ligation, which prevents the development of usual PSCV [41]. Patients may present with gross hematuria necessitating therapy [42].

#### *Pericholecystic varices*

**Fig. 13:**
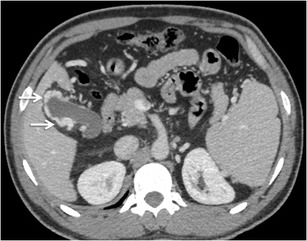
Axial CECT image showing multiple pericholecystic varices (*arrows*)

Pericholecystic varices refer to varices in or outside the wall of gallbladder in a pericholecystic location (Fig. 13) [3, 44]. They are present in approximately 12 % of patients with portal hypertension, but are more frequent in those with extrahepatic portal vein obstruction (30 %) [3]. The afferent veins are the cystic vein or a branch of the right portal vein, while the efferent drain into the hepatic vein, intrahepatic portal vein, or into systemic anterior abdominal wall collaterals [3].

#### *Bronchial varices*

Bronchial varices are speculated to develop through collateral channels which normally exist between the tracheal and oesophageal venous systems [45]. There have been only three case reports in English-language medical literature describing bronchial varices secondary to portal hypertension [45–47]. Two of these were in patients with alcoholic liver cirrhosis and oesophageal varices [46, 47], while the third was secondary to extrahepatic portal vein stenosis [45]. All the previously reported cases presented with hemoptysis. In one of them, the bleeding was massive and required portosystemic shunting and embolisation [47].

#### *Mesenteric collaterals*

Mesenteric collateral vessels may arise from the superior mesenteric vein (SMV) and inferior mesenteric vein (IMV) and ultimately drain into the IVC via the retroperitoneal or pelvic veins (also called the veins of Retzius) [48, 49]. In contrast to other portosystemic shunts, the veins of Retzius are often not dilated even in patients with portal hypertension, and hence are not well recognized. Various pathways of veins of Retzius are defined according to the receiving vein (mesenterico-gonadal/renal/caval or iliac)

#### *Retroperitoneal collaterals*

**Fig. 14:**
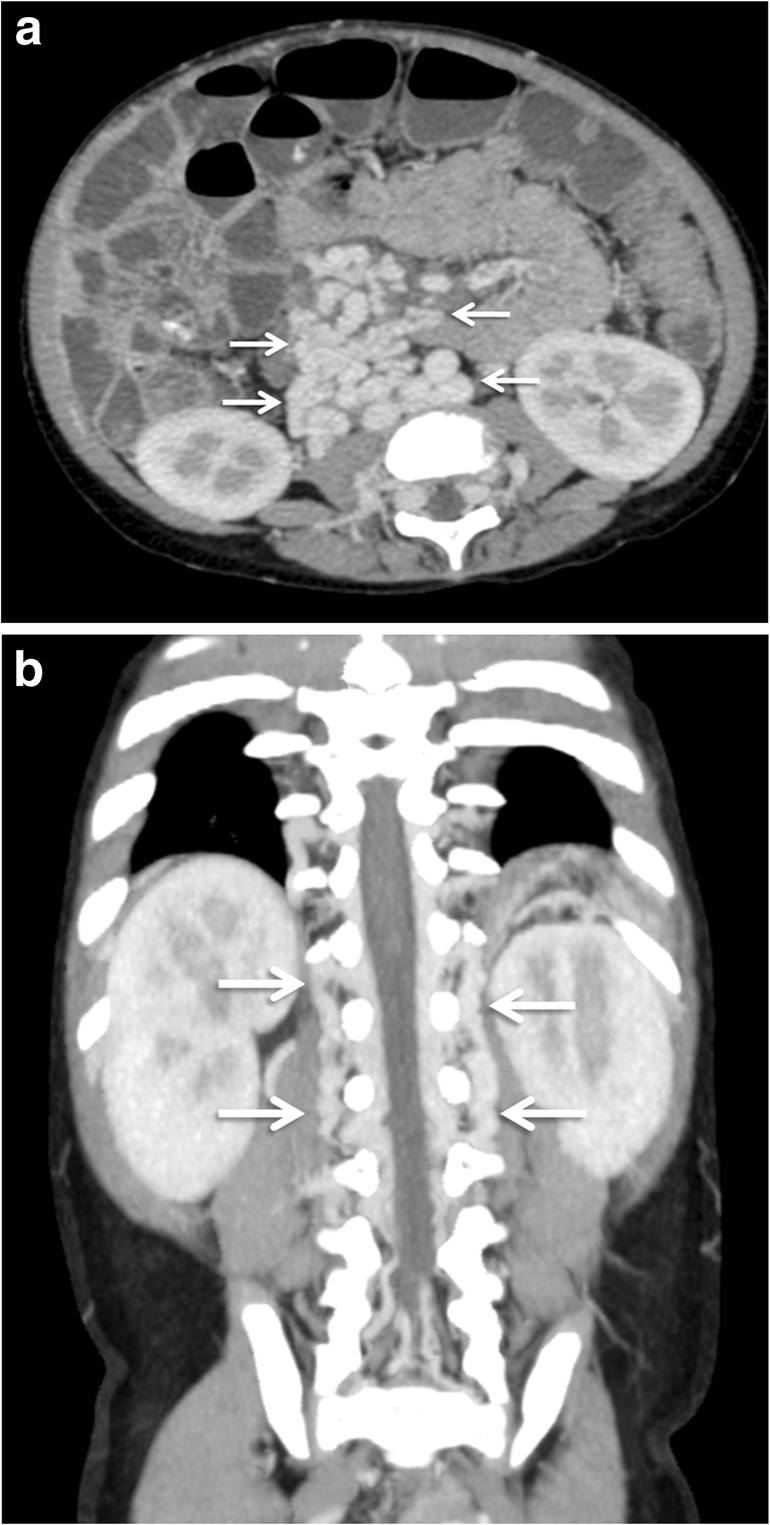
Coronal MIP image showing multiple retroperitoneal and paravertebral collaterals (*arrows* in a and b, respectively)

Retroperitoneal varices are thought to arise from the colic or mesenteric branches (veins of Retzius) and can occur anywhere in the retroperitoneum [3]. Collaterals may develop in the peripancreatic, perisplenic, perirenal, paravertebral (Fig. 14) and retrocaval area. Retroperitoneal collaterals may communicate with retrogastric varices or inferior phrenic vein. They may drain into the renal vein or directly into the IVC [3].

#### *Omental varices*

**Fig. 15:**
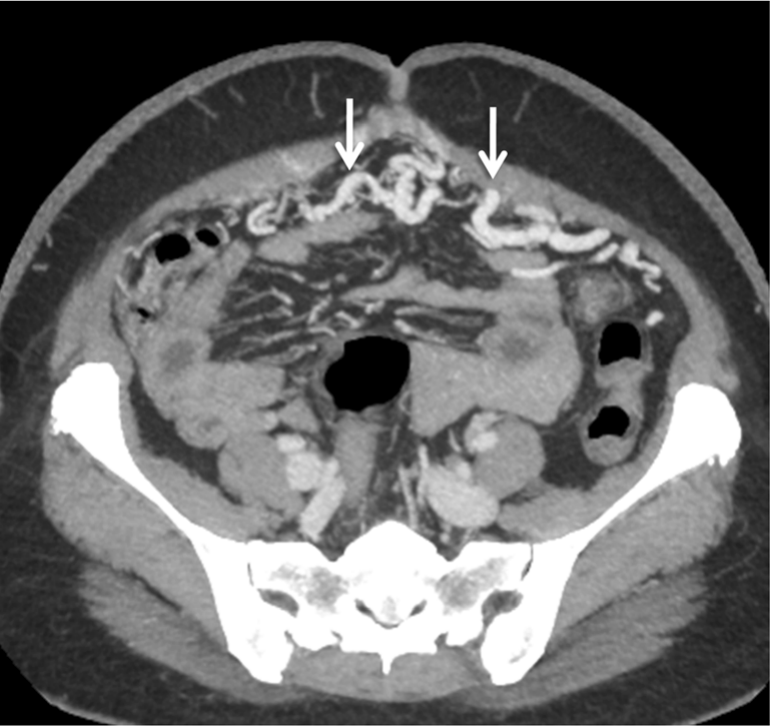
Axial MIP image showing multiple omental collaterals (*arrows*)

Omental collateral vessels are infrequently included in the lists of common portosystemic collateral vessels, presumably because they are not well visualized with angiography [6]. However, they can be effortlessly visualised on cross-sectional imaging (Fig. 15). Omental varices arise from the superior or inferior mesenteric veins and drain into the retroperitoneal or pelvic veins [3]. Occasionally they may drain into the gastro-oesophageal varices. The greater omentum, in contrast to the small bowel mesentery, has scanty vascular structures. In patients with portal hypertension and ascites, omental varices may mimic omental infiltration from carcinomatosis or peritonitis. More importantly, there have been reports of fatal episodes of bleeding from rupture of omental varices [50, 51]. Mortality remains high despite surgical correction of the bleeding underlining the importance of early detection and prompt surgical intervention.

#### *Anastomotic and stomal varices*

In the setting of chronic portal vein thrombus, collaterisation usually occurs through the hepaticoduodenal ligament, resulting in the formation of a portal cavernoma. However, in patients who have undergone previous hepatobiliary surgery, formation of the classical portal cavernoma can be precluded by the surgical dissection of preformed primitive vascular structures in the hepatoduodenal ligament [3]. In these patients, collateral channels can develop at unusual locations. Previously described entities include porto-portal varices in patients with enteroenteric anastomosis [52] and dilated communicating channels between jejunal veins and intrahepatic portal vein branches in patients with hepaticojejunostomy [53].

**Fig. 16:**
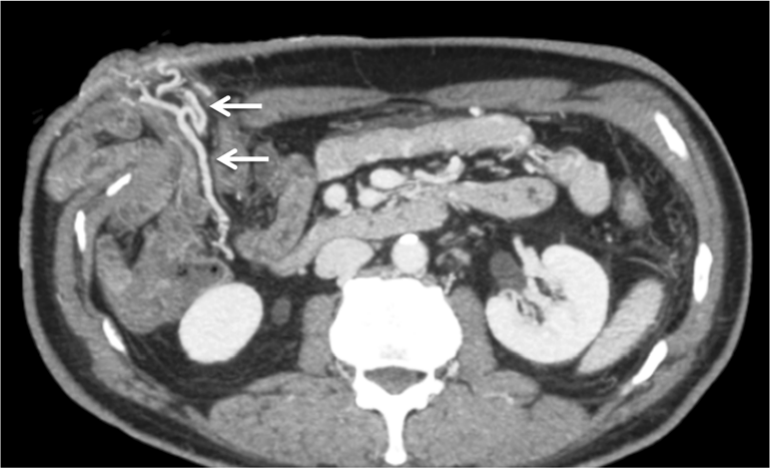
Axial MIP image showing stomal collaterals (*arrows*)

Surgically created bowel stomas create a communication between the high pressure portal venous network of the mesentery and the low pressure network of systemic veins in the abdominal wall, resulting in formation of stomal varices (Fig. 16). Approximately 50 % of patients with surgical digestive stoma in a context of portal hypertension have stomal varices [3].

#### *Rectal and perirectal varices*

**Fig. 17:**
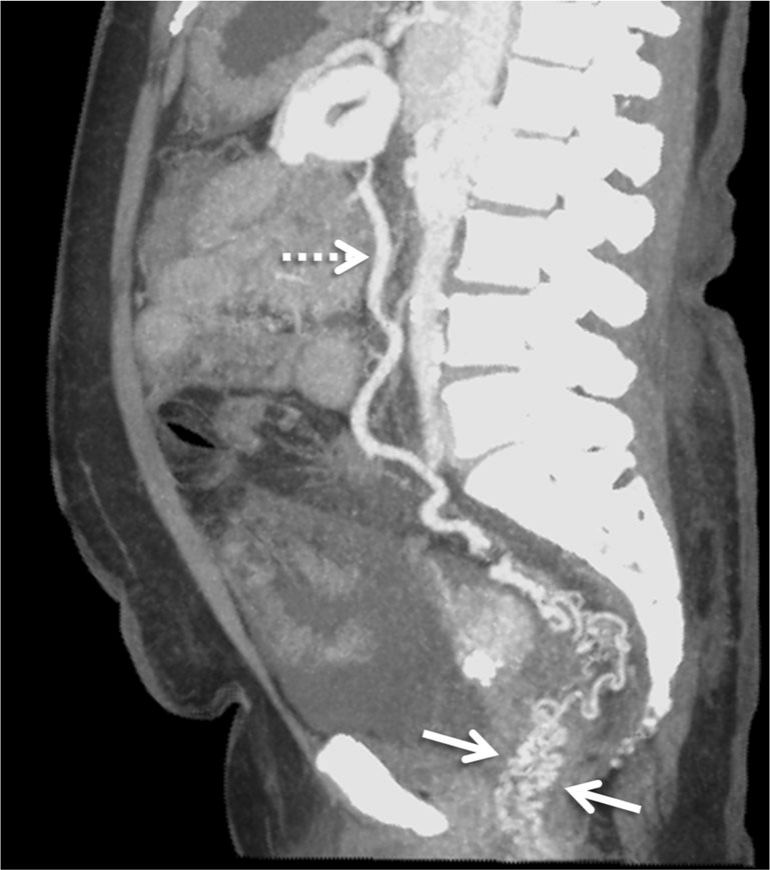
Sagittal MIP image showing multiple rectal collaterals (*arrows*) deriving their afferent supply from the IMV (*interrupted arrow*)

Rectal varices constitute a pathway for portal venous flow between the superior rectal veins of the inferior mesenteric system and the middle and inferior rectal veins of the iliac system and manifest as discrete dilated submucosal veins [3, 54] (Fig. 17). The superior rectal vein drains into the extrinsic rectal venous plexus (ERVP), which lies outside rectum. From the ERVP, the blood flows through the muscularis propria into the intrinsic rectal venous plexus (IRVP), which consists of a superior group lying in the rectal submucosa and an inferior group lying in the corresponding anal subcutaneous tissue. Rectal varices are formed from this superior group of submucosal veins of IRVP. The inferior group of IRVP forms the inferior rectal vein and contributes to formation of external haemorrhoids. Portal blood from both ERVP and IRVP drains into the systemic circulation through the recto-genital and inter-rectal portosystemic shunts.

For a surgeon considering an anorectal surgery, these collaterals are of special clinical concern, because an anorectal anastomosis through the inferior mesenteric vein can potentially cause catastrophic haemorrhoidal bleeding.

## Shunts

### Intra-hepatic shunts

**Fig. 18:**
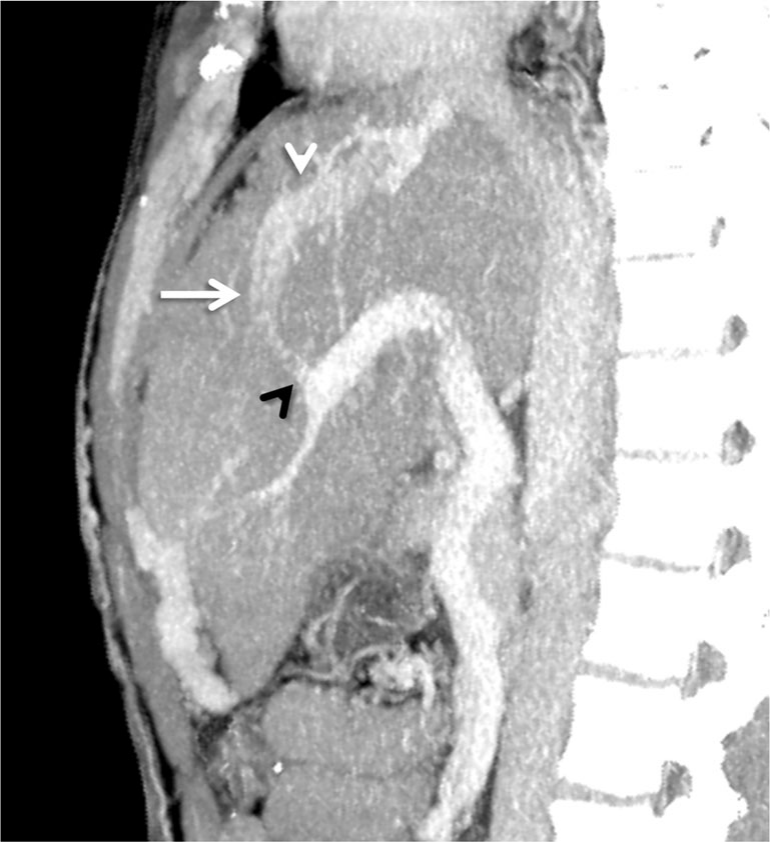
Sagittal MIP image showing an intrahepatic shunt (*arrow*) between the right portal vein (*black arrowhead*) and IVC (*white arrowhead*)

Intrahepatic portosystemic venous shunts (IPSVS) can be either congenital due to persistent embryonic venous anastomoses, or acquired due to cirrhosis, traumatic episodes, or rupture of a portal venous aneurysm into a hepatic vein. Based on the published case reports of IPSVS, Park et al. classified them into the following types: (1) single tubular shunt connecting the right portal vein to the inferior vena cava (most common type) (Fig. 18), (2) localized peripheral shunt in which one or more communications are found in a single hepatic segment, (3) portosystemic shunt through a portal vein ‘aneurysm’ and (4) multiple communications between peripheral portal and hepatic veins in several segments [55]. Their clinical significance lies in the fact that multiple or large IPSVS can result in the development of hepatic encephalopathy that might need to be treated by radiological intervention [56]. Also, this collateral pathway can preclude crucial procedures such as TIPS. In addition, type 1 and 3 IPSVS may mimic a hypervascular lesion like hemangioma on conventional CECT [10].

### Transhepatic shunts

Transhepatic PSCV involve intrahepatic branches of the portal vein that communicate with a systemic vein outside the liver, including the inferior vena cava, coronary vein, vertebral plexus, and hemiazygos vein [10]. In 1883, *Sappey* described accessory portal veins in the suspensory ligament entering the liver capsule through different locations, such as the vessels located at the falciform ligament through which the anterior parietal veins communicate with the left branch of the portal vein (Table 2) [57, 3]. These vessels play an important role in the origin of transhepatic portosystemic shunts [3, 10].

**Table 2:** Accessory portal veins described by *Sappey*

Site	Veins
Upper part of falciform ligament	Superior veins of Sappey
Lower part of falciform ligament	Inferior veins of Sappey
Bare area of liver	Diaphragmatic veins
Left triangular ligament	Left inferior phrenic vein and intercostal vein
Right triangular ligament	Right inferior phrenic vein
Gastrohepatic omentum	Cystic veins and branches of LGV

#### *Recanalised paraumbilical vein*

**Fig. 19:**
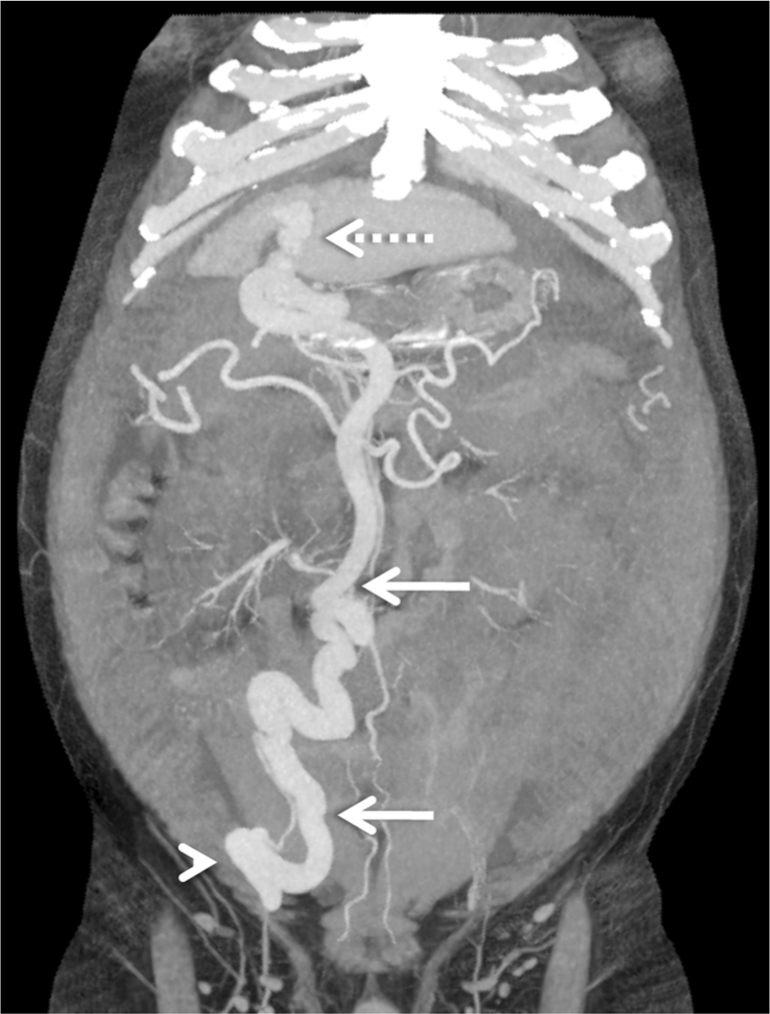
Coronal MIP image showing a dilated and tortuous recanalised paraumbilical vein (*solid arrows*) arising from the left portal vein (*interrupted arrow*) and draining into the right common iliac vein (*arrowhead*)

The paraumbilical veins, also called inferior veins of Sappey, are the most common type of transhepatic shunts that accompany ligamentum teres (obliterated left umbilical vein) in the falciform ligament [3]. Paraumbilical vessels may anastomose with the superior epigastric or internal thoracic veins and drain into the superior vena cava (SMV), or anastomose with the inferior epigastric vein and then drain into the IVC through the external iliac vein [48] (Fig. 19). The development of large recanalised paraumbilical vein has been found to prevent formation of bleeding oesophageal varices and to predispose to hepatic encephalopathy [58].

**Fig. 20:**
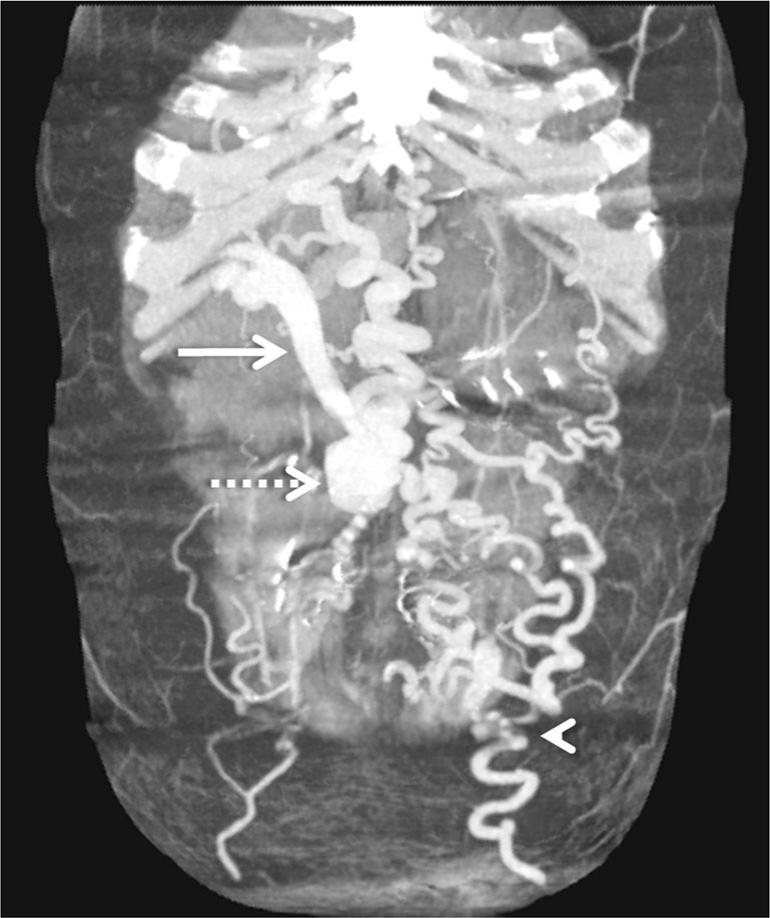
A giant recanalised paraumbilical vein draining via a large tuft of periumbilical varices (*interrupted arrow*) into the ipsilateral internal thoracic vein (*solid arrow*) as well as contralateral superficial epigastric vein (*white arrowhead*)

Occasionally, collateralisation can occur between a giant tortuous recanalised paraumbilical vein and the veins of the anterior abdominal wall (Fig. 20). This results in formation of a network of dilated periumbilical veins (‘medusa head’ appearance) known as Cruveilhier-Baumgarten syndrome manifesting clinically as abdominal wall bruit (the Cruveilhier-Baumgarten bruit) and a palpable thrill [58, 59].

In the presence of prominent anterior abdominal wall collaterals, a seemingly innocuous procedure like paracentesis could cause serious complications, if done without imaging guidance. Similarly, in the presence of abdominal wall or paraumbilical collaterals, even a simple hernia operation can become a dreaded procedure. Even with knowledge of a recanalised paraumbilical vein, the true extent and complexity may be underestimated without explicit information about its course and size. More recently, recanalised paraumbilical vein has been used as an access route for percutaneous embolisation of bleeding gastro-oesophageal and umbilical varices [60, 61].

#### *Right infradiaphragmatic shunt/ apex type shunt*

In the right infradiaphragmatic shunt, the collateral vein arising from a peripheral branch of left portal vein drains into the internal thoracic vein and the intercostal vein [10, 11]. This vein is also called the superior vein of Sappey [10]. The hepatofugal blood directed through this shunt into the internal thoracic vein reaches the right heart via the brachiocephalic vein and the SVC. In patients with SVC syndrome, contrast medium or isotopes injected into the arm go into the liver through this shunt, explaining the “hot” spot that is sometimes shown in the liver of these patients [10, 62].

#### Left infradiaphragmatic shunt/left triangular ligament shunt

**Fig. 21:**
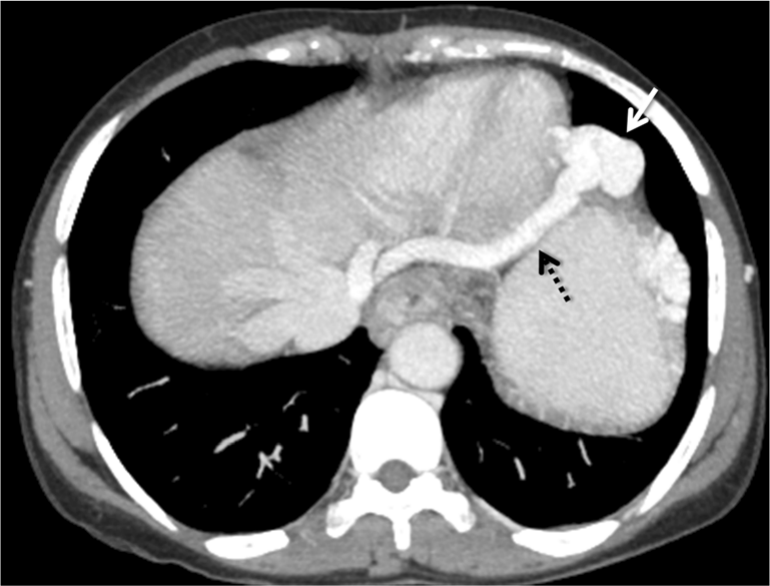
Left infradiaphragmatic shunt. A transhepatic shunt (*interrupted arrow*) wherein the collateral vein from the peripheral portal branch of the left lateral segment communicates with the left inferior phrenic vein at (*solid arrow*) the left triangular ligament

In this type of the shunt, the collateral vein arising from the peripheral portal branch of the left lateral segment communicates with the left inferior phrenic vein at the left triangular ligament, and drains into the IVC or the left renal vein through the intercostal vein or the left pericardiophrenic veins [10, 11] (Fig. 21).

#### *Right posterior portal branch-inferior vena cava (IVC) shunt*

Dilated collateral vessel arising from the right posterior portal vein runs across the posterior surface of the liver, forms a venous aneurysm outside the liver, and drains into the IVC directly or through the adrenal vein [10–12].

#### *Bare area shunt*

**Fig. 22:**
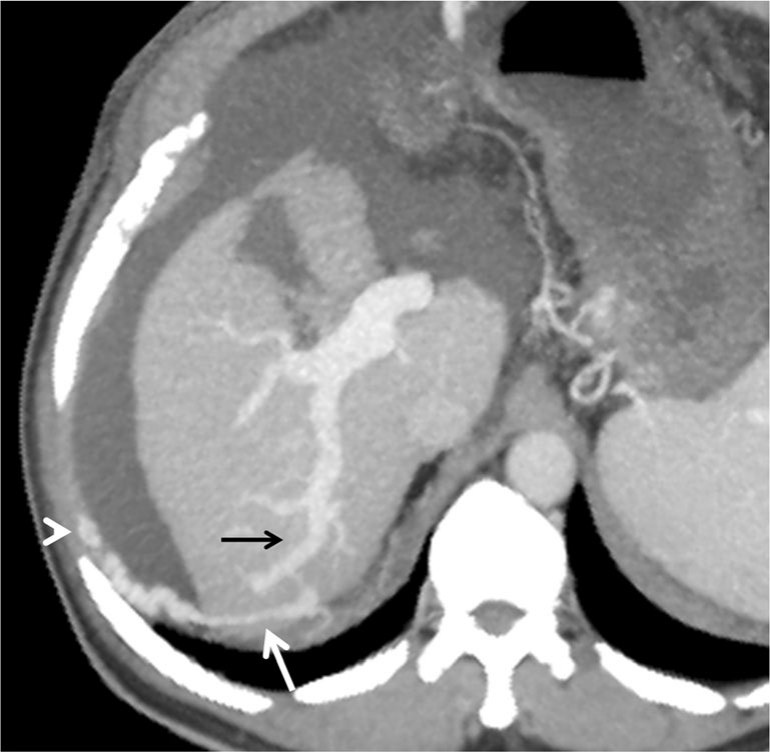
Bare area shunt. An intrahepatic shunt (*white arrow*) is seen arising from a peripheral branch of right portal vein (*black arrow*) and draining into the intercostal veins (*arrowhead*)

The peripheral branch of the right posterior portal vein runs across the surface of the liver and drains into the intercostal vein or the right inferior phrenic vein. In contrast to the right posterior portal vein type described previously, the vein does not show aneurysmal dilatation in the bare area type [10] (Fig. 22).

#### *Aberrant left gastric vein draining into the left portal vein*

**Fig. 23:**
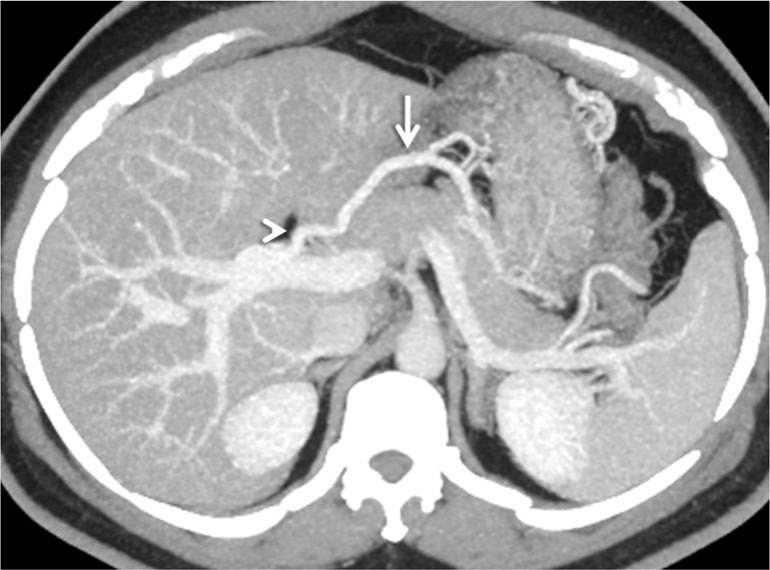
Aberrant left gastric vein (*arrow*) draining into the portal vein (*arrowhead*)

The left gastric vein usually drains the cardiac region of the lesser curvature of the stomach, and normally joins the spleno-portal confluence. The aberrant left gastric vein runs along the hepatogastric ligament and directly drains into the left portal vein [11], thus serving as hepatofugal collateral from the portal vein to systemic circulation (Fig. 23). This variant is of great relevance to the interventional radiologist as inadvertent leakage of sclerosant into the portal circulation during procedures such as B-RTO carries the risk of intrahepatic and extrahepatic portal venous thrombosis.

#### *Portoazygos shunt*

This refers to a communication between the main portal and the azygos veins. While congenital portoazygos shunts have been extensively described in certain dog breeds [63], it is rare in humans, with only a single case report describing this entity in a neonate with thoraco-abdominal duplication and absent intrahepatic portal vein [64]. Its description in association with liver cirrhosis and portal hypertension is also limited to a solitary case report. In this report, the shunt was seen between the posterior aspect of the main portal vein and the azygos vein along the right aspect of the thoracolumbar vertebrae [13]. It may be asymptomatic, but can cause hepatic encephalopathy or variceal bleeding. Treatment options include endovascular transvenous coil embolisation or surgical ligation [13].

### Extrahepatic shunts

#### *Gastrorenal and splenorenal shunts*

**Fig. 24:**
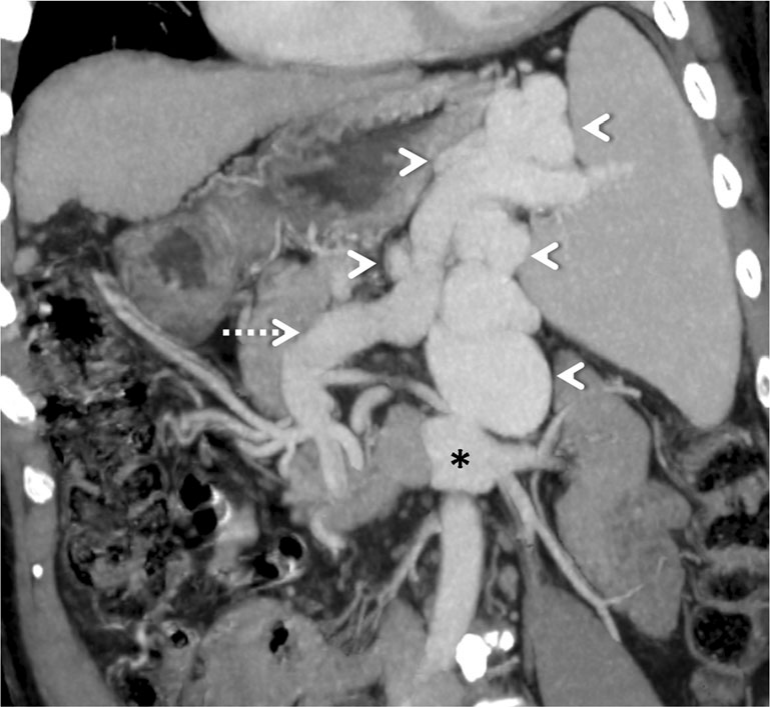
Coronal MIP image demonstrating a dilated and tortuous splenorenal shunt (*arrowheads*) communicating between the splenic vein (*interrupted arrow*) and the left renal vein (*asterisk*)

Gastric varices that usually drain into the oesophageal or para-oesophageal veins can occasionally drain into the left renal vein via a gastrorenal shunt [3, 25, 26] (Fig. 24). Among the extra-hepatic shunts, gastrorenal shunts are the most common [3]. They form generally through the lower branch of the left inferior phrenic vein, which opens directly into the renal vein (spleno-gastro-phreno-renal shunt), or through left adrenal vein [3]. The gastrocaval shunt drains through the upper branch of the inferior phrenic vein into the vena cava and is mostly continuous with the phrenicopericardial vein, which ultimately drains into the brachiocephalic vein [3].

Direct splenorenal shunts constitute a direct communication between the splenic vein and the left renal vein, sometimes through the splenic capsule. This type of direct portosytemic shunting is similar to the direct shunting of blood from posterior branch of the left gastric vein to para-oesophageal veins and azygos vein without formation of oesophageal varices.

Large spleno/gastro-renal shunts are often found in patients with recurrent or chronic hepatic encephalopathy, and B-RTOof these has shown good results in improving the patient’s neurological status [25, 65].

#### *Mesenterico-gonadal/renal/caval/iliac shunts*

**Fig. 25:**
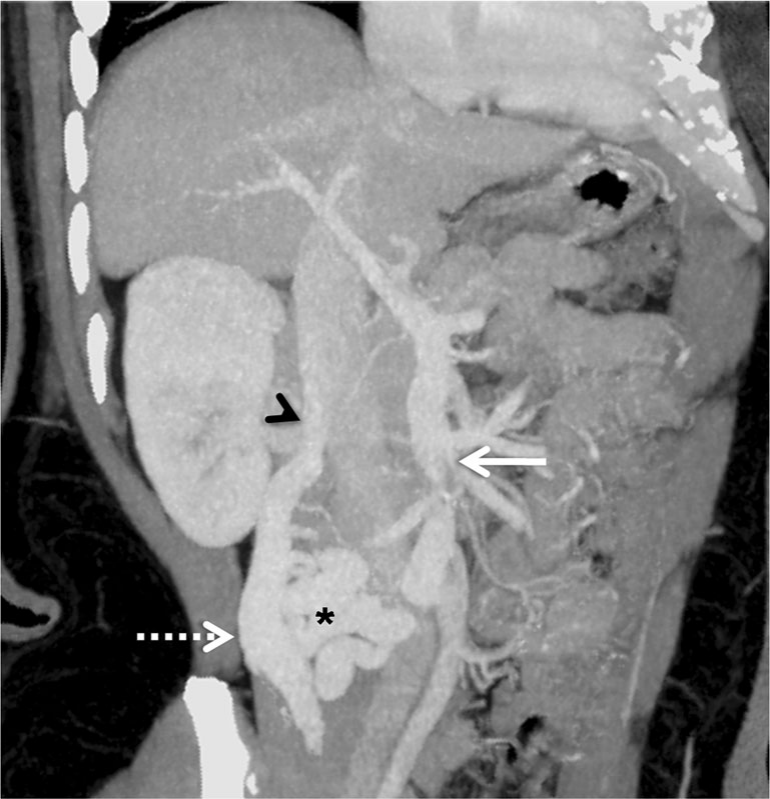
Coronal MIP image showing a mesocaval shunt (*interrupted arrow*) communicating between the SMV (*arrow*) and IVC (*arrowhead*) with a bunch of mesenteric collaterals (*asterisk*)

Mesenteric collaterals arising from the SMV and IMV may unusually drain into the systemic circulation via large shunts [48, 49]. Out of these, an ileocolic vein draining into the IVC or the right renal vein through the right gonadal vein (mesenterico- caval/gonadal varices) is the most frequently demonstrated pathway (Fig. 25) [3].

#### *Trans-splenic shunt*

**Fig. 26:**
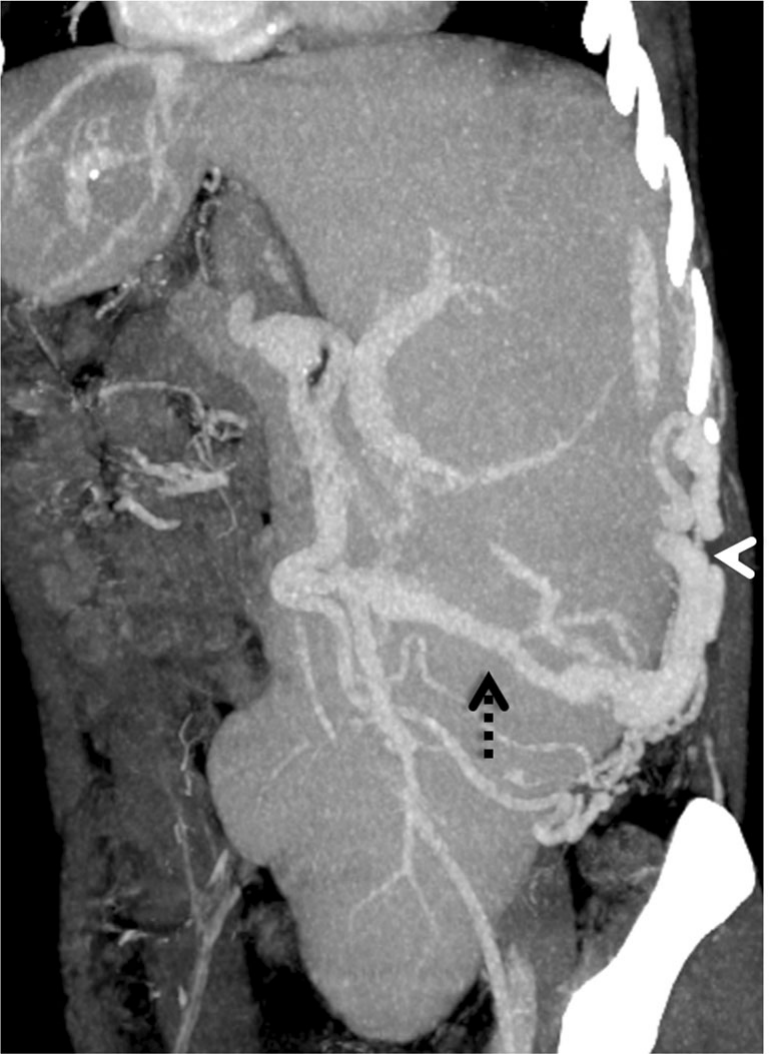
Coronal oblique MIP image showing a trans-splenic shunt (*interrupted arrow*) draining into the intercostal vein (*arrowhead*)

Trans-splenic shunts (Fig. 26) are extremely rare, with only two case reports describing this entity. One of these was in an adult patient with compensated cirrhosis [14]. The other was in a study of children with extrahepatic portal venous obstruction [66], which concluded that trans-splenic shunts were uncommon but that their presence is seen in children with cirrhosis and PHTN. Associated intra-splenic collaterals can occasionally be found [14].

#### Spleno-caval/phrenic/azygos shunt

The splenic vein or the perisplenic collaterals communicate with the hypogastric vein and ultimately drain into the IVC (splenocaval shunt). The splenic vein can also communicate with the left inferior phrenic vein, hemiazygos vein or the posterior abdominal wall veins [4].

#### Coronary/splenic-inferior pulmonary/inferior phrenic/intercostal veins

The left gastric vein or splenic vein communicates with the inferior pulmonary vein, pericardiophrenic vein or to intercostal vein [4].

PSCV developing in the setting of Budd-Chiari syndrome and extrahepatic portal venous obstruction are a separate topic and have not been discussed here.

## Conclusion

Unusual portosystemic collateral pathways are increasingly being encountered in the daily clinical practice. Since these could be an important cause of bleeding and hepatic encephalopathy, radiologists should be familiar with the imaging findings to effectively identify them and aid in therapeutic decision making. Also, pre-operative knowledge of the anatomy and course of these uncommon portosystemiccollaterals is essential for interventional radiologists and surgeons to avoid inadvertent vascular injury during the procedures.
